# Real-Time Monitoring of a Botulinum Neurotoxin Using All-Carbon Nanotube-Based Field-Effect Transistor Devices

**DOI:** 10.3390/s18124235

**Published:** 2018-12-03

**Authors:** Nam Hee Lee, Seung-Hoon Nahm, Insung S. Choi

**Affiliations:** 1 Department of Chemistry, KAIST, 291 Daehak-ro, Yuseong-gu, Daejeon 34141, Korea; n30237@kaist.ac.kr; 2 Energy Materials Metrology, Korea Research Institute of Standards and Science, 267 Gajeong-ro, Yuseong-gu, Daejeon 34113, Korea

**Keywords:** botulinum neurotoxin (BoNT), carbon nanotube (CNT), field-effect transistor (FET), microfluidic device, lateral flow immunoassay, stretchable sensor

## Abstract

The possibility of exposure to botulinum neurotoxin (BoNT), a powerful and potential bioterrorism agent, is considered to be ever increasing. The current gold-standard assay, live-mouse lethality, exhibits high sensitivity but has limitations including long assay times, whereas other assays evince rapidity but lack factors such as real-time monitoring or portability. In this study, we aimed to devise a novel detection system that could detect BoNT at below-nanomolar concentrations in the form of a stretchable biosensor. We used a field-effect transistor with a p-type channel and electrodes, along with a channel comprising aligned carbon nanotube layers to detect the type E light chain of BoNT (BoNT/E-Lc). The detection of BoNT/E-Lc entailed observing the cleavage of a unique peptide and the specific bonding between BoNT/E-Lc and antibody BoNT/E-Lc (Anti-BoNT/E-Lc). The unique peptide was cleaved by 60 pM BoNT/E-Lc; notably, 52 fM BoNT/E-Lc was detected within 1 min in the device with the antibody in the bent state. These results demonstrated that an all-carbon nanotube-based device (all-CNT-based device) could be produced without a complicated fabrication process and could be used as a biosensor with high sensitivity, suggesting its potential development as a wearable BoNT biosensor.

## 1. Introduction

Infectious diseases caused by pathogenic microorganisms (e.g., bacteria, viruses, or fungi) remain a significant cause of global mortality; specifically, such diseases took the lives of 9.2 million people in 2013, accounting for approximately 17% of all deaths worldwide. In particular, botulinum neurotoxins (BoNT), produced by anaerobic *Clostridium* bacteria, are considered the most poisonous protein products; these have 150 kDa molecular weight and seven structurally distinct serotypes (A to G) [1,2]. The lethal dose of BoNT for humans is estimated at approximately 1.3–2.1 ng/kg intravenously or intramuscularly, 10–13 ng/kg inhalationally, and 1.0 μg/kg (body weight in adults) orally [3,4]. Thus, neurotoxins, which are the causative factor of botulism, a rare but serious paralytic illness, are also considered a potential agent of bioterrorism. For example, the release and subsequent inhalation of 1 g of BoNT could lead to the deaths of more than one million people [5,6]. Additionally, food-borne botulism can become a public health issue when people get poisoned by eating contaminated food or drinking water. In this respect, issues of concern include how to avoid the disease in advance as well as how to rapidly prevent the spread of the pathogen. One of the best strategies is the development of real-time detection technologies having high sensitivity and quantitative analysis capability, which would enable us to prevent the diseases caused by neurotoxins or to screen individuals to allow for the proper treatment or quarantine them.

As a current gold standard, the live-mouse lethality assay is the most common method used to detect BoNT owing to its sensitivity (approximately 20 pg/mL), robustness, and simplicity [7]. However, this method requires long assay times (typically, 48 h), is expensive and laborious, and introduces an ethical dilemma regarding the use of laboratory animals. Alternative methods, such as mass spectrometric assays [8], enzyme-linked immunosorbent assays (ELISAs) [9,10,11], surface plasmon resonance [12], lateral flow immunoassay [13,14,15,16], high-performance liquid chromatography [17], and fluorescence resonance energy transfer [18] have successfully aimed at rapidity (within 20 min) and sensitivity (15–150 pg/mL). However, further research is still required to fulfill optimal criteria, such as real-time and label-free detection with rapidity, simplicity, and sensitivity including quantitative analysis and transportability.

Recently, field-effect transistor (FET)-based bioelectronics, involving the transduction of signals from the biological system to electrical signals at the bio-electronics interface, has been intensively investigated in various areas [19,20,21,22,23,24]. Owing to ultrasensitive detection and high-throughput, such real-time embedded systems potentially improve the fundamental understanding of biological phenomena and allow development of biomedical devices such as cardiac pacemakers, deep-brain stimulators, and blood glucose sensors. In particular, carbon nanotube (CNT)-based FET biosensors have received marked attention because of their excellent conductivity, durability for flexible and stretchable devices, exceptional aspect ratios, and various strategies available for carbon nanotube functionalization. For example, Li et al. [25] successfully characterized the complementary interaction of prostate with prostate antibody using a single-walled carbon nanotube-based FET. This system, presumably having a charge transfer sensing mechanism, afforded sensitivity comparable to that of metal oxide nanowires.

Therefore, we were encouraged to apply the all-carbon nanotube-based FET (all-CNT-based FET) to detect a botulinum neurotoxin with high sensitivity in real time and employed lateral flow assays (endopeptidase assay and immunoassay) to characterize the neurotoxin. CNTs employed in this study were aligned on an elastomer to improve device performance; additionally, electrodes and a semiconductor component were utilized to establish a flexible and stretchable biosensor.

## 2. Materials and Methods

### 2.1. Preparation of Protein Samples

The type E light chain of BoNT (BoNT/E-Lc) and antibody BoNT/E-Lc (Anti-BoNT/E-Lc) were obtained from the Department of Biological Sciences and Laboratory of Immunology and Infectious Diseases in KAIST [26]. BoNT/E-Lc (50 kDa) was in solution form at 7.4 μM, dissolved in 50 mM Tris-buffer solution (pH 7), whereas the antibody was a monoclonal immunoglobulin G (mAb IgG, 160 kDa) in solution form at 8.6 μM in 10 mM phosphate-buffered saline (PBS). We purchased the 29-mer peptide with a minimal essential SNAP-25 domain from Peptron (Daejeon, Korea). The 29-mer peptide sequence is as follows: N-GL Aib AA GGDMG NEIDT QNRQI DRIME KADK-C.

In the experiment using the peptide, a mixture of 10 mM HEPES (pH 7.4), 5 mM dithiothreitol (DTT), 10 mM Tween 20, and 20 mM ZnCl_2_ was used as the reaction buffer solution. For the antibody-antigen interaction, a mixture of 50 mM Tris-buffer solution, 5 mM DTT, and 10 mM Tween 20 was used as the reaction buffer solution. All materials used in the experiment were purchased from Sigma-Aldrich (St. Louis, MO, USA). To confirm the BoNT/E-Lc activity for product peptides and antibodies, BoNT type A light chain (BoNT/A-Lc; 0.07 μg/μL, List Biological Laboratories, Inc., Campbell, CA, USA) was used.

### 2.2. Preparation of Sensor Cover with a Microfluidic Channel

A polydimethylsiloxane (PDMS) film with a micro-sized channel was produced using an established method [27]. Briefly, SU-8 photoresist (MicroChem Corp., Newton, MA, USA) was spin-coated onto a washed silicon wafer and soft baked for 20 min at 95 °C. Photolithography was then utilized to create a pattern forming a fluidic channel on the SU-8 layer, followed by baking at 95 °C for 10 min and developed (SU-8 developer; MicroChem Corp.), completing the PDMS mold. The PDMS (Sylgard 184; Dow Corning, Midland, MI, USA) film was made by pouring the PDMS precursor into the mold and allowing it to harden at room temperature for >12 h. The PDMS precursor was a 1:20:2 mixture of cross-linking reagent, base, and hexane. Finally, the hardened PDMS film was removed from the mold and used as the sensor cover with a microfluidic channel (length 6 mm, width 500 μm, depth 250 μm).

### 2.3. Fabrication of the All-CNT-Based FET Device

A spinnable CNT synthesized via the chemical vapor deposition method was incorporated for the CNT layer used as the electron channel, source, and drain in the device (Figure S1) [28,29]. The obtained aligned CNT layer (length 20 mm, width 15 mm, and height 0.1 mm) was attached on the PDMS film to be used as a sensor substrate. The size of the attached CNT channel was 8 mm in length and 1 mm in width. The CNT channel obtained the electrical characteristics suitable for the sensor by high voltage and O_2_ plasma.

After applying 50 V high voltage for 5 s using a high-voltage wideband amplifier (Tabor Electronics Ltd., Haifa, Israel), O_2_ plasma treatment (power = 100 W, flow rate = 50 sccm, pressure = 70 Pa) was applied for 10 min. The CNT layer, to be used as the electrical leads, was attached to both ends of the high voltage/plasma-treated CNT channel. Then, 1 × 2 mm^2^ gold foil was attached by pressing onto these ends for contact with the probe measuring the electrical properties.

### 2.4. CNT Surface Modification

To demonstrate BoNT/E-Lc detection, the product peptides and Anti-BoNT/E-Lc were attached on the high voltage/plasma-treated CNT channel. First, to achieve immobilization of the peptides on the CNT surface, we used a non-covalent bonding method via the pyrene group of a succinimidyl ester [25,30]. To prepare the succinimidyl ester solution (in dimethyl formamide (DMF)), *N*-hydroxysuccinimidyl pyrenebutanoate (Sigma-Aldrich) was used, and the prepared 6 mM succinimidyl ester solution was added dropwise into the CNT channel. After 3 h, the molecules that did not react were rinsed away with DMF. After washing the CNT surface with 10 mM HEPES buffer solution (pH 7.4), 0.8 μM peptide was incubated for 6 h. Determination of the concentration of the appropriate peptide for the experiment is shown in Figure S2. To achieve real-time detection of the specific recognition between BoNT/E-Lc and Anti-BoNT/E-Lc, the CNT channel with Anti-BoNT/E-Lc was prepared using the same process. The CNT surface containing succinimidyl ester was washed with 50 mM Tris-buffer solution (pH 7.4) and was exposed to 10 nM Anti-BoNT/E-Lc for 6 h. All of the aforementioned treatment processes were performed inside the PDMS walls with a circular hole (diameter 3 mm) on top of the CNT channel.

### 2.5. Electrical Measurement

Two-probe electrical measurement was performed for testing the sensor’s electrical properties and protein sensing. Electrical measurements were performed by a voltage source (7651 programmable DC source, Yokogawa, Tokyo, Japan), and a multimeter (34401A digital multimeter, Hewlett Packard, California, USA). 

Conductance vs. time data were recorded every 0.1 s with buffer and target protein flowing through the microfluidic channel. The reaction buffer solution flow began 1 min after the conductance vs. time recording started. The buffer solution was made to flow from the start of the experiment. When the sensor conductance stabilized, protein flowed in place of the buffer solution. All experiments were performed with a 0.01 μL/min flow rate using a syringe pump, using reaction buffer solutions suitable for each experiment; e.g., for observing peptide cleavage, 10 mM HEPES (pH 7.4) was used, whereas 50 mM Tris-buffer solution (pH 7.4) was used for observing specific bonding with the antibody. However, both solutions included 5 mM DTT and 10 mM Tween 20. To increase the activity of BoNT/E-Lc, BoNT/E-Lc samples were immersed in water with a constant temperature of 37 °C. Conductance vs. time recording was performed with the sensor placed on the heater to maintain a temperature of 37 °C. BoNT/E-Lc detection using antibody-antigen-specific binding was performed under room temperature, and the electrical signals were measured while attached to the vial surface (diameter 70 mm). A comparative experiment using BoNT/A-Lc was conducted using the same experimental conditions as the experiment targeting BoNT/E-Lc. The room temperature at which the experiment was performed was an average of 24 °C. 

### 2.6. Characterization of CNT Surface 

Atomic force microscopy (AFM, INNOVA-LABRAM HR800, Horiba Jobin Yvon, Kyoto, Japan) was used for surface analysis of CNTs. X-ray photoelectron spectroscopy (XPS, Sigma Probe, Thermo VG Scientific, Waltham, MA, USA) was also performed to identify functional groups on the functionalized CNT surface.

## 3. Results and Discussion

### 3.1. Characteristics of the All-CNT-Based FET Device

**Figure 1:**
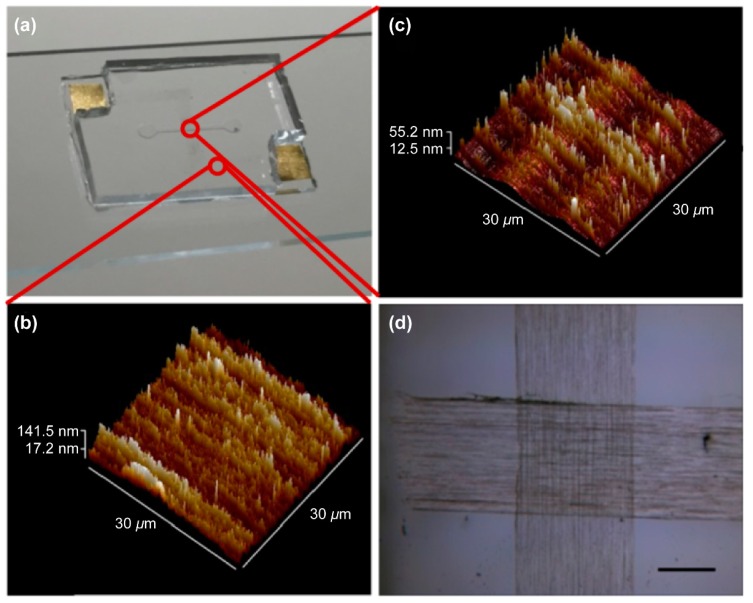
Image of the manufactured all-CNT-based device. (**a**) Actual appearance of the CNT-based device. (**b**) AFM image of the electrode composed of CNT layers. The electrodes consist of a CNT layer with an average thickness of 90 nm. (**c**) AFM image of the CNT layers used as active channels of the device. The channel has an average thickness of 40 nm and CNTs in the channel have a density of 0.03 CNTs/μm^2^. (**d**) Microscopic image showing contact between electrodes and channels made up of CNT layers; scale bar = 500 μm.

All-CNT-based devices for detecting BoNT/E-Lc were constructed using the production process shown in Figure S3b. To increase device sensitivity, we applied electrical breakdown and O_2_ plasma to the CNT channel. Changes in the aligned CNT layers before and after high voltage and plasma treatment designed for CNT surface functionalization were confirmed using AFM and electrical characterization. As shown in Figure 1, CNT layer thickness decreased after treatment. Moreover, CNT layer’s electrical properties changed from primarily metallic to p-type semiconducting (Figure S4). This might be ascribed to the majority of metallic CNTs being burned away upon application of voltage [31], as well as oxygen atoms binding to many surface defects formed on the surface of CNTs from O_2_ plasma treatment, indicating that the positive charges of the oxygen atoms had been transferred to the CNTs [32,33]. We measured the electrical characteristics of a CNT-based FET device that underwent electrical breakdown and O_2_ plasma treatment using a back gate with SiO_2_ (120 nm) as a dielectric layer. For this experiment, a CNT-based FET device built on a Si/SiO_2_ substrate was employed.

**Figure 2:**
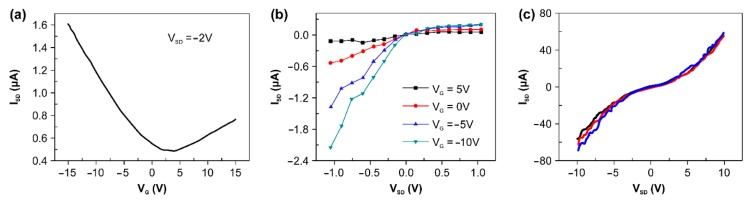
Electrical properties of the all-CNT–based device. (**a**) Transfer characteristics of the CNT device measured at V_SD_ = −2 V. (**b**) Output characteristics measured with V_G_ from −10 to 5 V in 5 V steps. (**c**) Output current as a function of V_SD_ measured at V_G_ = 0 for 3 different bending conditions (radius of curvature: 15 mm (black line), 35 mm (red line), relax (blue line). Insert: image of CNT device attached to a curved surface.

Figure 2a,b show the results of measuring the output current change according to the various gate voltages and of the change of current with the voltage change. The electrical measurement results confirmed that the device fabricated was operated by holes; i.e., Figure 2a,b demonstrate that the CNT-based FET device formed a p-type channel.

Based on the measurement results, we next attempted to obtain the effective device mobility ($\mu_{\mathrm{eff}}$), which can be estimated by:

(1) $$\mu_{\mathrm{eff}}=\frac{\partial I_{D}}{\partial V_{G}}\;\frac{L}{\mathrm{WC}V_{D}},$$

where I_D_ is the drain current, V_G_ is the gate voltage, L is the channel length, W denotes a channel width, and V_D_ denotes a drain voltage [34]. In the equation for the effective device mobility, C is the gate capacitance and can be estimated from:

(2) $$C=\frac{\varepsilon \varepsilon_{0}}{t},$$

where ε is the dielectric constant of the gate dielectric SiO_2_, ε_0_ is the vacuum permittivity, and t is the thickness of the gate dielectric. The effective device mobility estimated based on these equations was ≈ 1.3 × 10^4^ cm^2^V^−1^s^−1^ (L = 8 mm, W = 1 mm, V_SD_ = −2 V).

**Figure 3:**
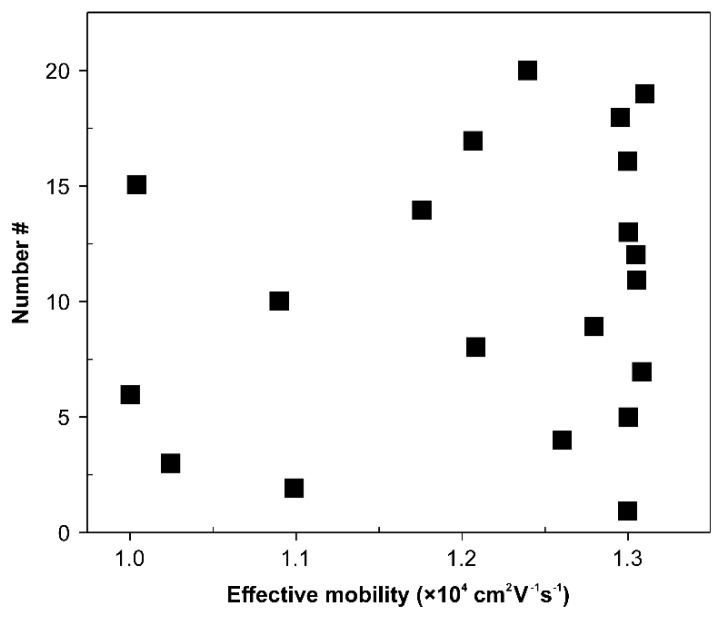
Distribution of the effective field effect mobility of 20 all-CNT-based FETs. When the size of the CNT channel is 8 mm in length and 1 mm in width, the average mobility is 1.2 × 10^4^ cm^2^V^−1^ s^−1^.

The results of the effective device mobility for 20 FETs built on a Si/SiO_2_ substrate are summarized in Figure 3. Their average mobility was (1.2 ± 0.1) × 10^4^ cm^2^V^−1^s^−1^.

Considering the characteristics of a flexible and sticky device, we observed electrical characteristics of the device in the bent conformation. By attachment to a vial surface with varying diameter, changes in current according to a source-drain voltage ranging from −10 to +10 were measured (Figure 2c). We found that there was no significant difference in device resistance between attachment onto a surface of flat glass or on vials (diameters of 30 and 70 mm).

We produced an all-CNT-based device using spinnable CNT layers and confirmed its functionality. Simultaneously, we achieved our goal of creating the basis for BoNT/E-Lc detection.

### 3.2. Real-Time Monitoring of the Interaction between Specific Peptides and BoNT

BoNT/E-Lc has the unique property of cleaving the specific peptide bonds of SNAP-25 because BoNT/E-Lc is a zinc-metalloprotease that hydrolyzes the bond between arginine (R) 180 and isoleucine (I) 181 in the SNAP-25 sequence [21,22,35]. Using these properties, we attempted to detect BoNT/E-Lc on the previously prepared all-CNT-based FET devices. Previously, we created a microfluidic device by attaching the sensor cover with the microfluidic channel (created via the process shown in Figure S3) to a device with 29-mer product peptides. Real-time sensing of peptide cleavage was performed by flowing varying concentrations of BoNT/E-Lc on CNT device that contained the unique peptides. This experiment was carried out under cleavage conditions of the peptides; i.e., pH 7.4 and 37 °C.

**Figure 4:**
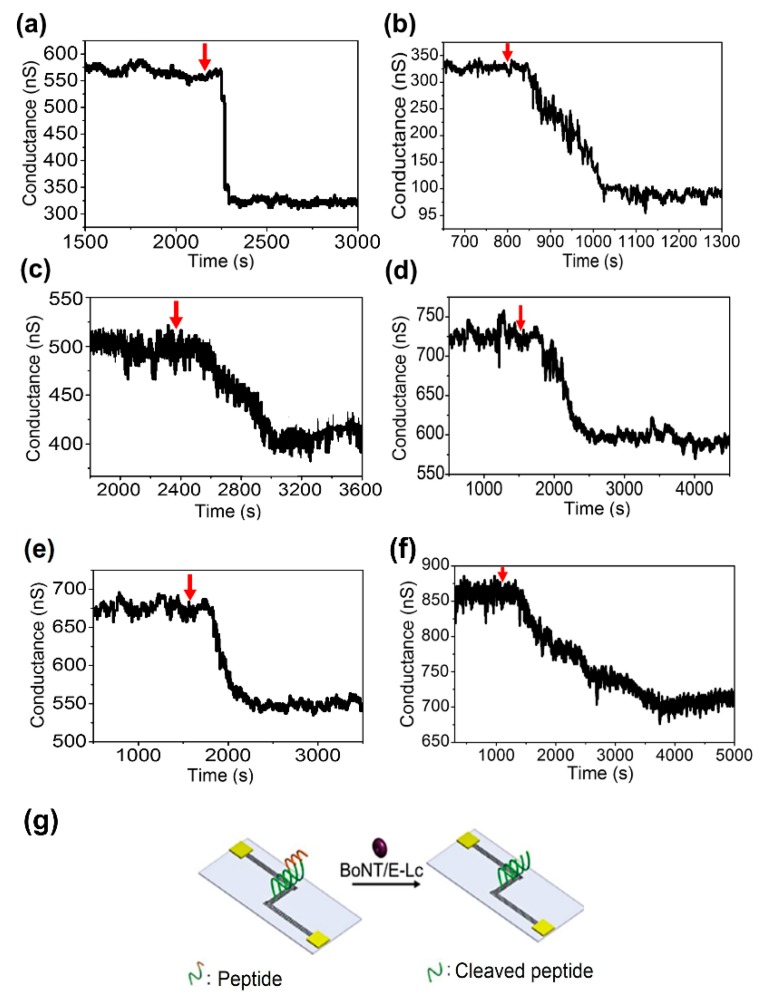
Real-time monitoring of the interaction between specific peptides and BoNT/E-Lc. Measurement of electrical signal changes owing to specific reaction between varying concentrations of BoNT/E-Lc and unique peptides. (**a**) 5 nM BoNT/E-Lc; (**b**) 3 nM BoNT/E-Lc; (**c**) 1 nM BoNT/E-Lc; (**d**) 0.3 nM BoNT/E-Lc; (**e**) 0.1 nM BoNT/E-Lc; (**f**) 60 pM BoNT/E-Lc; (**g**) Schematic of the cleavage of the 29-mer peptide by BoNT/E-Lc. The red arrow in the figure indicates the injection of the target molecules.

**Figure 5:**
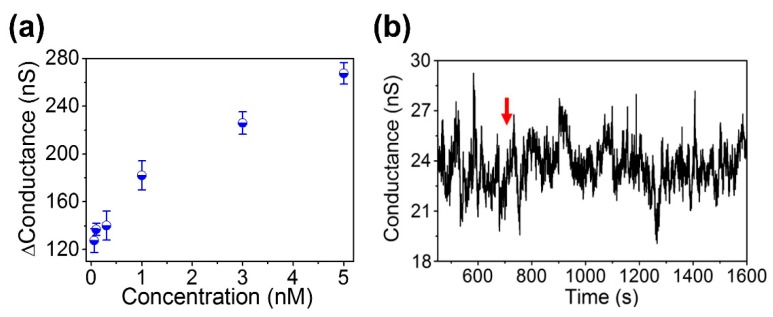
(**a**) Comparing electrical signals at six different concentrations of BoNT/E-Lc; (**b**) determination of selectivity in specific interactions using 3 nM BoNT/A-Lc. The red arrow in the figure indicates the injection of the target molecules.

The electrical response of the sensor at six different BoNT/E-Lc concentrations (5 nM, 3 nM, 1 nM, 0.3 nM, 01. nM, and 60 pM) is shown in Figure 4. When different BoNT/E-Lc concentrations were injected, the device conductivity decreased compared to when only the buffer solution was allowed to flow. This results were attributed to BoNT/E-Lc cleaving seven amino acids from the peptide C-terminus. In other words, the reduction in the conductivity of the sensor at constant concentration is caused by the reduction of intrinsic charges of peptides, which had a local effect on the electrical field of device. The experimental relationships between the conductivity change of the sensor and the BoNT/E-Lc concentration are shown in Figure 5a. From the test results, we also found that the higher the concentration of BoNT, the greater the change in conductivity. This finding implies that higher concentrations of BoNT produced more peptide cleavage.

The times required from injection of BoNT/E-Lc to the saturation of conductivity were 3 min at 3 nM and 38.3 min at 60 pM (Figure 4b,f). However, from injection of the target proteins, electrical signal changes were observed within 1 min at 3 nM and within 5 min at 60 pM. However, compared with Figure 4b,f, the time required for saturation of changed conductivity showed a 3-fold difference, whereas conductivity change was only approximately 10 nS. 

Finally, we confirmed the selectivity of BoNT/E-Lc for the unique peptide. 3 nM BoNT/A-Lc was selected as the target substance, as it can cleave SNAP-25 similarly to BoNT/E-LC [35]. As shown in Figure 5b, no electrical signal change events based on BoNT/A-Lc injection were observed. Comparison of these results to Figure 5b indicated that BoNT/A-Lc did not have any effect on product peptide cleavage and that only BoNT/E-Lc exhibited reactivity toward the peptides. Whereas BoNT/E-Lc hydrolyzes the Arg180-Lie181 bond of SNAP-25, BoNT/A-Lc disrupts the SNAP-25 Gln197-Arg198 bond and thus does not appear to have reactivity toward the product peptides.

In conclusion, specific reactions between the product peptides and BoNT/E-Lc were demonstrated, confirming the utility of the devised system for this purpose.

### 3.3. Observation of Specific Recognition between BoNT/E-Lc and Anti-BoNT/E-Lc

**Figure 6:**
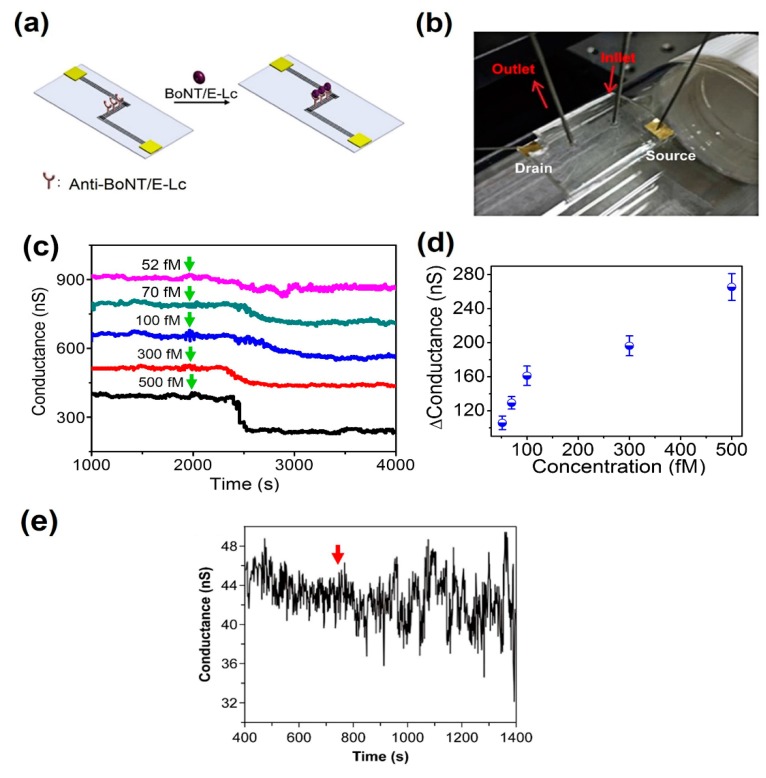
Sensing of the specific binding between BoNT/E-Lc and Anti-BoNT/E-Lc. (**a**) Illustration of BoNT/E-Lc binding with Anti-BoNT/E-Lc. (**b**) Optical image of BoNT/E-Lc detection through the microfluidic channel in the bent state. (**c**) Real-time detection of BoNT/E-Lc with varying concentrations (green arrow: BoNT/E-Lc solution injection). (**d**) Sensor response at various concentrations of BoNT/E-Lc. (**e**) Determination of selectivity for BoNT/E-Lc with CNT-based sensors using BoNT/A-Lc (red arrow: 500 fM BoNT/A-Lc injection).

We also conducted experiments for BoNT/E-Lc detection using antigen-antibody interaction. This method can be performed at room temperature and is adapted in many biosensors owing to its relatively straightforward mechanism, which constitutes specific binding between the epitopes found on the antigen and the binding site on the antibody. This experiment was initiated by attaching the PDMS cover with a microfluidic channel onto the CNT-based device with the Anti-BoNT/E-Lc. With the manufactured microfluidic device in the bent state, the conductivity change was checked in real time with 500, 300, 100, 70 and 52 fM BoNT/E-Lc (Figure 6a–c). When varying concentrations of flowed BoNT/E-Lc solution, the conductivity was seen to decrease within 1 to 5 min in comparison to when flowing only the buffer solution (Figure 6c). Figure 6d shows the relationship between the response of the sensor and the BoNT/E-Lc concentration. As can be seen from the figure, the relationship is not completely linear. However, the value of the conductance change increases with increasing BoNT/E-Lc concentration. We surmise this might be because BoNT/E-Lc, which has a positive charge at pH 7.4, binds to antibodies, thereby imposing a localized effect at the electric field on the CNT device.

BoNT/A-Lc was also used to determine the selectivity of CNT-based sensors. Upon 500 fM BoNT/A-Lc injection, no changes in electrical signal were observed (Figure 6e). Although the noise level increased as BoNT/A-Lc passed through, no tendency for change in conductivity was seen. In summary, using our novel CNT-based device, we successfully detected BoNT/E-Lc at concentrations below 55 fM, the known lethal dose (LD_50_) of BoNT. In particular, we also showed that detection of biomolecules through the microfluidic channel is possible even in the bent state.

### 3.4. Surface Analysis of Carbon Nanotubes Using X-Ray Photoelectron Spectroscopy (XPS)

The XPS was used to investigate the interaction of the molecules that occurred on the surface of the CNTs. In this experiment, the main task of XPS is the observation of nitrogen introduced by biomolecules. The binding energy of nitrogen is ~400.4 eV [36].

**Figure 7:**
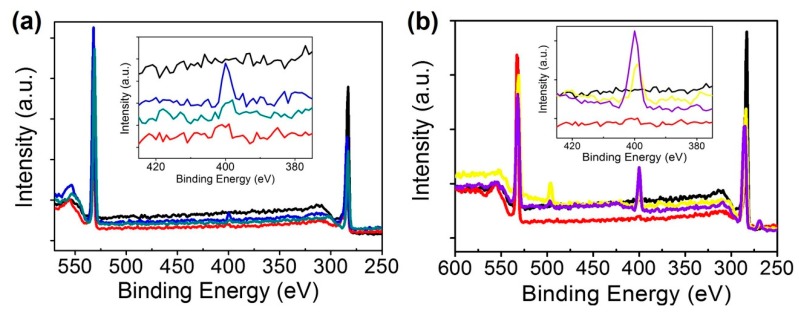
Results of XPS measurements of carbon nanotube surfaces undergoing a series of chemical treatments. Chemical changes on the surface of carbon nanotubes were confirmed based on the presence and content of carbon, oxygen, and nitrogen. (**a**) Identification of specific interaction between BoNT and peptides. (**b**) Confirmation of specific recognition between Anti-BoNT and BoNT. Each color has the following meanings. Black: bare CNTs, red: succinimidy ester (SE)/CNTs, blue: unique peptides/SE/CNTs, greed: Cleaved peptides/SE/CNTs, yellow: Anti-BoNT/SE/CNTs, violet: BoNT/Anti-BoNT/SE/CNTs.

Figure 7a shows the results of a carbon nanotube surface undergoing a biochemical reaction in which a portion of the 29 mer-peptide is cleaved by BoNT/E-Lc. The interaction between BoNT/E-Lc and specific peptides was clearly determined by XPS analysis. According to the results, the atomic percentage of 2.2% for N1s possessed by peptide-functionalized CNTs was reduced to 1.5% by BoNT. A decrease in the atomic concentration of N1s means that a shortened peptide was produced by BoNT. On the other hand, the specific recognition between BoNT/E and Anti-BoNT/E leads to an increase in the atomic concentration of N1s (Figure 7b). The atomic percentage of 6.84% for N1s in CNTs with Anti-BoNT increased to 13.23% as a result of antigen‒antibody-specific binding. Also, In the measurement results, peaks corresponding to carbon (C1s) and oxygen (O1s) with binding energy of ~283.15 eV and ~532.45 eV were found.

## 4. Conclusions

We produced a new type of all-CNT-based device using a simple production process. Using this device, we attempted to detect BoNT/E, which is emerging as one of the candidates of biochemical warfare. By monitoring the cleavage of a unique peptide and the specific binding between BoNT/E and Anti-BoNT, we could confirm the high sensitivity of the all-CNT device. Through multiple BoNT/E detection experiments, we showed the potential for expanding the biosensor industry as well as the possibility of producing a biosensor that can detect biomaterials within a short time to counteract possible biological weapons. Although developing a device with consistently high performance is beset by difficulties, our results may provide the basis for the fabrication of a CNT-based device that will not require an alignment process of CNTs or the transfer of grown CNTs. Furthermore, we suggest that it may also enable the fabrication of a wearable biosensor.

## Supplementary Materials

The following are available online at http://www.mdpi.com/1424-8220/18/12/4235/s1, Figure S1: Images of spinnable CNTs synthesized for the CNT layer, Figure S2: Determination of product peptide BoNT/E (0.1 nM) was used to determine the change in thickness of the peptide, which had a specific reaction duration of 15 min, Figure S3: Schematic diagram of the production process for the all-CNT–based microfluidic device designed to detect botulinum toxin, Figure S4: Electrical properties of the CNT device before and after electrical breakdown and O2 plasma treatment.

## Patents

As a result of this study, the sensor for detecting botulinum neurotoxin using spinning carbon nanotubes was registered as a domestic patent whose registration number is 10-1903255-0000.
