# Supplementary material for: Real-Time Monitoring of a Botulinum Neurotoxin Using All-Carbon Nanotube-Based Field-Effect Transistor Devices
Source: Sensors (Basel). 2018 Dec 3;18(12):4235. doi: 10.3390/s18124235 (PMC6308983; doi:10.3390/s18124235)
Supplement: Supplementary file 1 [file sensors-18-04235-s001.pdf]

Supplementary Material

Real-time monitoring of a Botulinum neurotoxin using all-carbon nanotube-based field-effect transistor devices

Experimental procedure: Preparation of protein samples

The BoNT/E-Lc-specific monoclonal antibody was prepared by a standard hybridoma technique. Splenocytes were isolated from mice after immunization with BoNT/E-Lc twice at 1-week intervals. Hybridoma cells that secrete monoclonal antibodies to BoNT/E-Lc were produced by fusing splenocytes and myeloma cells (Sp2/0-Ag14; Korean Cell Line Bank). The hybridomas were selected and maintained, and their supernatants were screened for the presence of anti-BoNT/E-Lc antibodies by indirect ELISA. Briefly, microplates were coated with BoNT/E-Lc (5 μg/mL) and monoclonal hybridoma culture supernatants were added. Subsequently, anti-mouse Ig-HRP and substrates were added sequentially. The result was analyzed using an ELISA reader and 2 clones (clone 4 and clone 12) producing antibodies against BoNT/E-Lc with the highest optical density were selected. The selected hybridoma cells were seeded into the peritoneal cavity of Balb/c mice, and ascites fluid containing monoclonal antibodies was obtained. Monoclonal antibodies were purified by IgG column chromatography.

Supplemental Figures and Legends

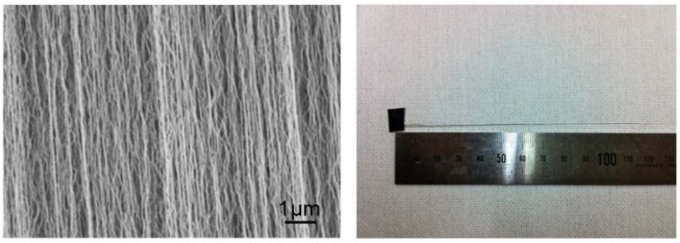

Fig. S1. Images of spinnable CNTs synthesized for the CNT layer. Double-sided tape was used to pull on the end portion of the vertically grown multi-walled CNT forest to form the CNT alignment.

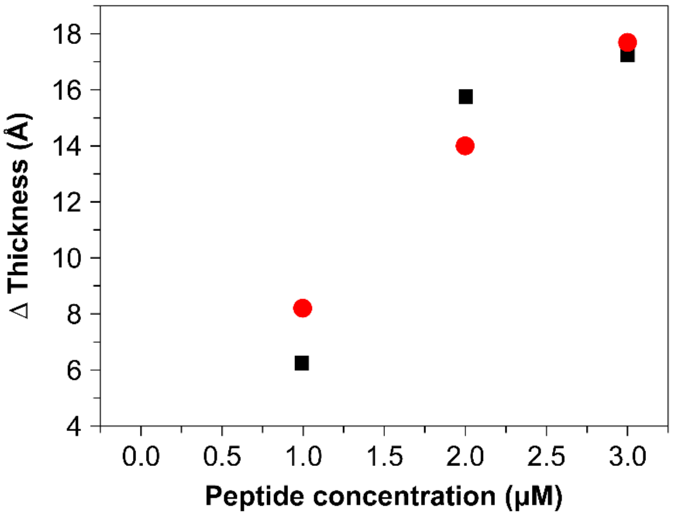

Fig. S2. Determination of product peptide BoNT/E (0.1 nM) was used to determine the change in thickness of the peptide, which had a specific reaction duration of 15 min. The peptide concentrations used were 0.1, 0.5, 1, and 3 μM. The change in peptide thickness was the greatest at a concentration of 1 μM. This concentration was designated as the standard concentration for optimal reaction condition, and the detection experiment was conducted at ± 0.2 of this concentration.

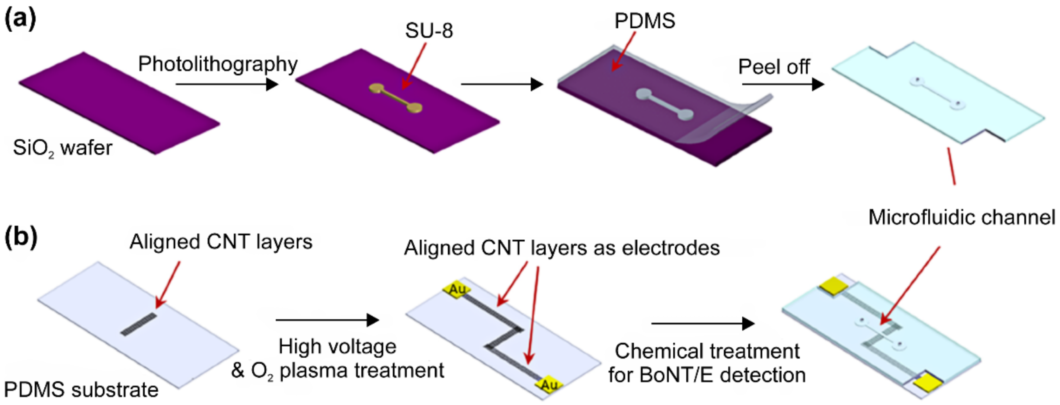

Fig. S3. Schematic diagram of the production process for the all-CNT–based microfluidic device designed to detect botulinum toxin. (A) Production of the PDMS cover with the microfluidic channel. (B) Production of the all-CNT–based device.

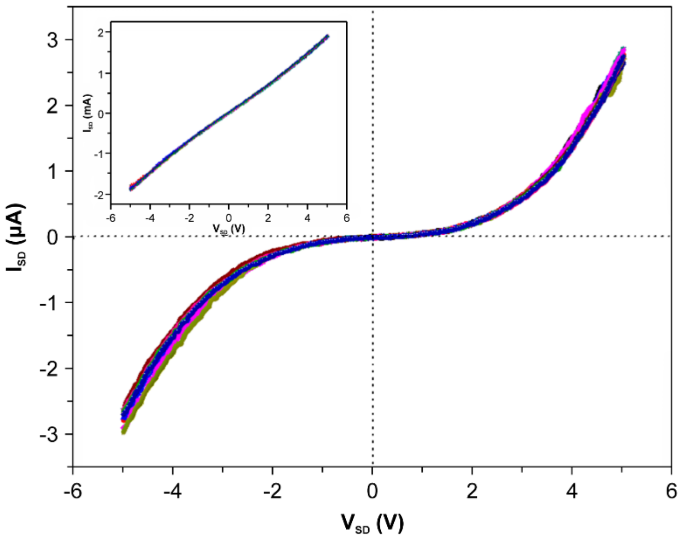

Fig. S4. Electrical properties of the CNT device before and after electrical breakdown and O_2 plasma treatment. The CNT channel on the device with metallic properties (insert) was treated to impart semiconducting properties. The voltage-current curves of devices fabricated by employing spinnable carbon nanotube arrays exhibit conductor-specific properties in the form of liner. On the other hand, semiconductor-specific voltage-current curves that do not conduct current in any section of the voltage are observed in carbon nanotube-based devices that have undergone electrical breakdown / O_2 plasma treatment.
